# Supplementary material for: Evaluation of Smallpox Vaccination Coverage and Attitude towards Monkeypox Vaccination among Healthcare Workers in an Italian University Hospital
Source: Vaccines (Basel). 2023 Nov 22;11(12):1741. doi: 10.3390/vaccines11121741 (PMC10747083; doi:10.3390/vaccines11121741)
Supplement: Supplementary file 1 [file vaccines-11-01741-s001.zip › vaccines-2611935-supplementary.pdf]

Table S1. Extract from the risk assessment (RA) for the different wards.

Department	Type of Exposure	Risk Assessment
Virology	possible direct exposure to the virus	From low to high risk
Emergency room	assistance to infected cases (doctors and health assistants)	From low to moderate risk
Emergency medicine	assistance to infected cases (doctors and health assistants)	From low to moderate risk
Infectious diseases	assistance to infected cases (doctors and health assistants)	From low to moderate risk
Proctological surgery	assistance to infected cases (doctors and health assistants)	From low to moderate
Dermatology	assistance to infected cases (doctors and health assistants)	From very low to low risk
Otolaryngology	assistance to infected cases (doctors and health assistants)	From very low to low risk
Urology	assistance to infected cases (doctors and health assistants)	From very low to low risk
Andrology	assistance to infected cases (doctors and health assistants)	From very low to low risk
Pediatric ED	assistance to infected cases (doctors and health assistants)	From low to moderate risk
Pediatrics	assistance to infected cases (doctors and health assistants)	From very low to low risk
Dentistry	assistance to infected cases (doctors and health assistants)	From very low to low risk

Table S2. Year of birth and department of HCWS who did not complete the self-declaration.

	Born since 1979	Born before 1979	*p*
Department			
• Infectious diseases	2 (22.2%)	7 (77.8%)	
• Emergency medicine	69 (59.5%)	47 (40.5%)	0.012
• Virology	0 (0%)	5 (100%)	
• Proctological surgery	2 (50%)	2 (50%)	
Infectious diseases	2 (22.2%)	7 (77.8%)	0.044
Not infectious diseases	71 (56.8%)	54 (43.2%)	
Emergency medicine	69 (59.5%)	47 (40.5%)	0.003
Not emergency medicine	4 (22.2%)	14 (77.8%)	
Virology	0 (0%)	5 (100%)	0.013
Not virology	73 (56.6%)	56 (43.4%)	
Proctological surgery	2 (50%)	2 (50%)	0.855
Not proctological surgery	71 (54.6%)	59 (45.4%)	

The undersigned ________________________ sex ________

born in ________________ (______) the ______/______/______

Job duty ________________________________

department ________________ Phone number ________________

e-mail ________________________________

Aware that anyone who makes false statements is punished pursuant to the Penal Code and the special laws on the subject, pursuant to and by effect of art. 46 Presidential Decree No. 445/2000

DECLARES

- to have been vaccinated for SMALLPOX:

YES □ NO □

If positive,

- NUMBER OF DOSES:
  1 □ 2 □ I do not remember □
- possession of any certification: YES □ NO □

FINALLY DECLARES

In the case of the vaccination offer, do you intend to undergo vaccination?
YES □ NO □

Pisa, ________________

The declarer

____

Figure S1. Self-declaration translated into English.
